# Macrophage phagocytosis of SARS-CoV-2-infected cells mediates potent plasmacytoid dendritic cell activation

**DOI:** 10.1038/s41423-023-01039-4

**Published:** 2023-05-30

**Authors:** O. García-Nicolás, A. Godel, G. Zimmer, A. Summerfield

**Affiliations:** 1 Institute of Virology and Immunology (IVI), Sensemattstrasse 293, 3147 Mittelhäusern, Switzerland; 2 Department of Infectious Diseases and Pathobiology, Vetsuisse Faculty, University of Bern, Bern, Switzerland; 3 Multidisciplinary Center for Infectious Diseases, University of Bern, Bern, Switzerland

**Keywords:** SARS-CoV-2; COVID-19, Monocyte-derived macrophages, Plasmacytoid dendritic cell, Interferon-α, Inflammatory cytokines, Phagocytes, Dendritic cells, Innate immunity

## Abstract

Early and strong interferon type I (IFN-I) responses are usually associated with mild COVID-19 disease, whereas persistent or unregulated proinflammatory cytokine responses are associated with severe disease outcomes. Previous work suggested that monocyte-derived macrophages (MDMs) are resistant and unresponsive to SARS-CoV-2 infection. Here, we demonstrate that upon phagocytosis of SARS-CoV-2-infected cells, MDMs are activated and secrete IL-6 and TNF. Importantly, activated MDMs in turn mediate strong activation of plasmacytoid dendritic cells (pDCs), leading to the secretion of high levels of IFN-α and TNF. Furthermore, pDC activation promoted IL-6 production by MDMs. This kind of pDC activation was dependent on direct integrin-mediated cell‒cell contacts and involved stimulation of the TLR7 and STING signaling pathways. Overall, the present study describes a novel and potent pathway of pDC activation that is linked to the macrophage-mediated clearance of infected cells. These findings suggest that a high infection rate by SARS-CoV-2 may lead to exaggerated cytokine responses, which may contribute to tissue damage and severe disease.

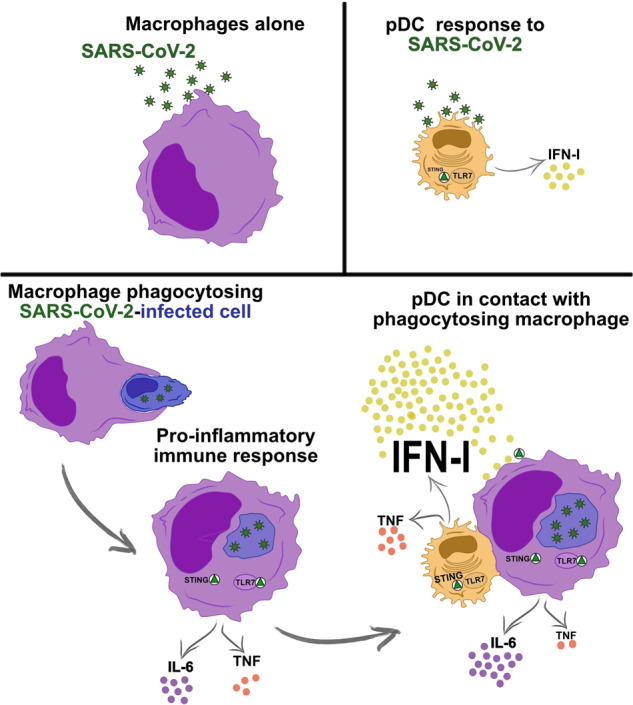

## Introduction

Severe acute respiratory syndrome coronavirus-2 (SARS-CoV-2), which was identified in late 2019 as the etiological agent of coronavirus disease 2019 (COVID-19), caused an unprecedented pandemic and global health crisis [1]. While the majority of individuals suffer from mild respiratory disease, some patients develop life-threatening inflammatory multiorgan disease that is associated with high morbidity and mortality rates. It has been proposed that innate immune responses represent a key determinant of COVID-19 disease severity [2]. In fact, in patients suffering from severe COVID-19, massive infiltration of proinflammatory immune cells, including activated macrophages, was detected in infected lung tissue. Immune cell infiltration along with excessive local inflammatory cytokine production were linked to exacerbated lung damage, which finally resulted in acute respiratory distress syndrome (ARDS) [3, 4]. The cytokine storm associated with severe COVID-19 is characterized by high levels of proinflammatory cytokines, including interleukin-1β (IL-1β), IL-2, IL-6, tumor necrosis factor (TNF), C-X-C motif chemokine ligand 10 or granulocyte-macrophage colony-stimulating factor (GM-CSF), among others [5, 6]. Importantly, macrophages are one of the main sources of proinflammatory cytokines such as IL-6 and TNF and were found to play a central role in the pathology of severe COVID-19 [7, 8]. Indeed, one of the most threatening forms of COVID-19 has been termed “macrophage activation syndrome” [9].

Although SARS-CoV-2 proteins were detected in macrophages from the lymph nodes of COVID-19 patients [10], human monocyte-derived macrophages (MDMs) do not express viral proteins or produce infectious virus following inoculation with SARS-CoV-2 in vitro [8, 11]. Furthermore, MDM infection did not induce detectable proinflammatory cytokines at the protein level [11]. Nevertheless, a recent report showed that an abortive, angiotensin-converting enzyme 2 (ACE2)-independent infection of macrophages with SARS-CoV-2 resulted in the induction of proinflammatory cytokine transcripts [12].

In addition to proinflammatory cytokines, the interferon type I (IFN-I) response is a key factor in determining the outcome of COVID-19. Early robust IFN-I responses seemed to control viral replication and were frequently associated with mild disease, while autoantibodies to IFN-I were associated with the development of severe COVID-19 [13–16]. Moreover, aberrant signaling of TLR and IRF, both important players in the IFN-I response, were also linked to severe COVID-19 [16]. As a result of poor IFN-I responses, continuous virus replication can cause aberrant and prolonged inflammation characterized by sustained secretion of multiple proinflammatory cytokines [3, 4, 17]. Plasmacytoid dendritic cells (pDCs) are responsible for the production of most systemic IFN-I during the early stages of viral infections [18, 19]. Like other RNA viruses, pDCs sense SARS-CoV-2 RNA through endosomal TLR7, resulting in IFN-I and inflammatory cytokine responses [20–25]. Moreover, TLR2-mediated sensing of SARS-CoV-2 envelope proteins has been reported to induce IL-6 production [24]. In patients suffering from moderate and severe COVID-19, the percentage of pDCs in peripheral blood is decreased, most likely due to the recruitment of these cells to the site of infection. In fact, pDCs can be found in the lung tissue of COVID-19 patients, including those with severe symptoms [17, 25–27]. In addition to the positive effects mediated by pDC responses, the observations that tissue damage in long-term COVID-19 patients correlated with sustained IFN-I secretion also support the role of pDCs in pathogenesis [28–30].

The current study was conducted to understand the mechanisms of macrophage and pDC activation by SARS-CoV-2. Based on our previous results, which demonstrated that SARS-CoV-2 is unable to directly activate human MDMs [11], we hypothesized that activation of these cells may require bystander interaction with SARS-CoV-2-infected cells. Furthermore, based on the observation that pDCs are often more efficiently activated by infected cells than by direct virus contact [31–35], we also investigated this scenario for SARS-CoV-2. The present study proposes a novel pDC-macrophage axis that is stimulated by the phagocytosis of virus-infected cells. Therein, tight transcellular interactions result in high levels of pDC-derived IFN-I and TNF, as well as enhanced levels of macrophage-derived IL-6.

## Materials and methods

### Cells

Vero cells (E6 lineage, African Green monkey kidney epithelial cells) were cultured in Dulbecco’s minimal essential medium (DMEM) supplemented with 10% fetal bovine serum (FBS), nonessential amino acids, penicillin‒streptomycin and HEPES (all cell culture reagents from Thermo Fisher Scientific, Zug, Switzerland, used as recommended by the manufacturer). A549 cells (adenocarcinomic human alveolar basal epithelial cells) stably transfected with ACE2 and transmembrane protease serine 2 (TMPRSS2), defined here as A549AT, were purchased from Invivogen (Toulouse, France), certified as Mycoplasma-free and cultured in minimal essential medium (MEM) supplemented with 10% FBS, 0.5 μg/ml puromycin (Invivogen) and 300 μg/ml hygromycin B (Invivogen).

Peripheral blood mononuclear cells (PBMCs) were isolated from buffy coats by density gradient centrifugation using Ficoll-Paque™ PLUS (1.077 g/l; GE Healthcare Life Sciences, Dübendorf, Switzerland). MDMs were generated as previously described [11]. Briefly, monocytes were isolated by magnetic cell sorting with anti-CD14 magnetic beads (Miltenyi Biotech, Bergisch Gladbach, Germany) and cultured in 24-well plates in “MDM medium” (RPMI 1640 supplemented with 10% FBS, GlutaMAX and penicillin‒streptomycin) supplemented with macrophage colony-stimulating factor (M-CSF, 100 ng/ml; Miltenyi Biotec). After differentiation, the cells were cultured in “MDM medium” with or without GM-CSF (100 ng/ml; Miltenyi Biotec) for an additional 18 h before use. For some experiments, MDMs were treated with different inhibitors as indicated below.

Human pDCs were isolated from PBMCs using the “Plasmacytoid Dendritic Cell Isolation Kit II” and LS columns (both from Miltenyi Biotech) following the manufacturer’s instructions. Immediately after isolation, as specified below, 5 × 10^4^ pDCs were cocultured in 500 µl of MDM medium with other cells; for controls, pDCs were seeded alone in 200 µl in 96-well U bottom plates and stimulated with virus or TLR ligands.

### Viruses

SARS-CoV-2 (SARS-CoV-2/München-1.1/2020/929) was kindly provided by Daniela Niemeyer, Marcel Müller, and Christian Drosten (Charité, Berlin, Germany) and propagated in Vero E6 cells in DMEM supplemented with 2% FBS and nonessential amino acids at 37 °C. For titrations, tenfold serial dilutions were added to Vero E6 cells, and after 72 h, the cytopathic effect was evaluated. Viral titers were expressed as 50% tissue culture infective dose per ml (TCID_50_/ml). Vesicular stomatitis virus (VSV) encoding green fluorescent protein (GFP) and expressing the C-terminally truncated spike protein of SARS-CoV-2 (VSV*ΔG-S_Δ21_) was previously described [36].

### Infection and stimulation of cells

Cell lines (Vero E6 cells or A549AT) and primary cells (MDM or pDC) were inoculated with SARS-CoV-2 or VSV*ΔG-S_Δ21_ using a multiplicity of infection (MOI) of 1 TCID_50_/cell as indicated for each experiment. Supernatants of uninfected cells were used as mock controls. After 1.5 h of incubation, the virus inoculum was removed, and the cells were washed three times with phosphate buffered saline (PBS, 37 °C) and incubated in medium (described above) at 37 °C in a 5% CO_2_ atmosphere. For certain experiments, primary cells were stimulated with different TLR ligands as positive controls for the induction of proinflammatory cytokines: lipopolysaccharide (LPS; 1 µg/ml; Sigma‒Aldrich, Buchs, Switzerland), polyinosinic-polycytidytic acid (poly I:C; 10 μg/ml; Sigma‒Aldrich), CpG oligodeoxynucleotide 2006 (CpG2006; 5 μg/ml; InvivoGen) or transfected with cyclic 3’3’-cGAMP ([G(3’,5’)pA(3’,5’)p], cGAMP; 10 μg/ml; InvivoGen) in 1 to 1 dilution with TransIT-LT1 transfection reagent (Mirus).

For inhibitory experiments, MDMs were pretreated with each compound 2 h prior to the experiment and kept in the cultures. Ruxolitinib (10 µM; InvivoGen) was used as a JAK1/2 inhibitor to block IFN-I signaling. The oligonucleotide IRS 661 (5′-TGCTTGCAAGCTTGCAAGCA-3′; 1.7 µM; TIB-Molbiol, Berlin, Germany) was used as a TLR7 inhibitor. The small molecule H-151 (1-(4-ethylphenyl)-3-(1H-indol-3-yl) urea; 10 µM; Sigma‒Aldrich) was used as a STING pathway inhibitor. Appropriate dimethyl sulfoxide (DMSO; Sigma‒Aldrich) controls were used for all inhibitors.

### Cocultures

To evaluate phagocytosis of infected cells, cell lines (Vero E6 or A549AT) were infected with either SARS-CoV-2 or VSV*ΔG-S_Δ21_, cultured for 18 h and harvested using trypsin (Sigma‒Aldrich). Immediately after harvesting, cells were labeled with the “Cell Trace Violet Cell Proliferation Kit” (CTV, Thermo Fisher Scientific) following the manufacturer’s instructions. Then, 5 × 10^5^ labeled cells were added to each well of MDM cultures in 24-well plates or different vessels if specified. Cocultured cells were incubated for 2 h at 37 °C, floating cells were removed, and all wells were washed three times with warm PBS. Finally, 500 µl of MDM medium was added to each well, and the cells were kept in culture for 24 h. MDM incubated with either Vero E6 or A549AT, as described above, are referred to throughout the manuscript as “MDM-φ-VeroE6” or “MDM-φΑ549ΑΤ”, respectively. For the phagocytosis inhibitory experiments, MDMs were pretreated for 2 h with Cytochalasin D (CytD; 5 µM; CAYMAN chemical company INC, Ann Arbor, MI, USA) and readded during coculturing with SARS-CoV-2-infected Vero E6 cells. Appropriate DMSO (0.05% v/v; Sigma‒Aldrich) controls were included. As previously described [25], the influence of IFN-α on MDM activation was tested by supplementing the medium with either 700 IU/ml recombinant human interferon alpha (IFN-α; α 2b; Gibco) or conditioned medium from pDCs stimulated for 24 h with SARS-CoV-2 (10% v/v). After 24 h at 37 °C in a 5% CO_2_ atmosphere, either SARS-CoV-2-infected or mock-treated Vero E6 cells were added to allow phagocytosis. Where indicated, MDMs were pretreated with ruxolitinib for 2 h prior to stimulation with IFN-α. After 2, 24, 48 or 72 h of coculturing, supernatants were harvested and stored at −70 °C, and cells were detached using TrypLE™ Select (Gibco) for 20 min at 37 °C.

To evaluate pDC activation, infected Vero E6 cells (as above) were cocultured with MDMs for 2 h (as above) and then washed three times with warm PBS before the addition of 5 × 10^4^ pDCs per well in 500 µl of MDM medium. In some experiments, pDCs were physically separated from MDMs by seeding them into 24-well plate Transwell inserts with 1 μm diameter pores (Corning, Sigma‒Aldrich). Alternatively, the physical interaction between pDCs and MDMs was disrupted by adding an anti-human CD11a monoclonal antibody (10 µg/ml, clone HI111, Thermo Fisher Scientific) during the coculture of MDMs with pDCs. In other experiments, TLR7 (IRS 661) or STING pathway (H-151) inhibitors were added to the MDM-pDC coculturing medium as indicated before. For all pDC experiments, 18 h of incubation at 37 °C in 5% CO_2_ was used.

### Determination of phagocytosis and infected cells

After coculture, MDMs were harvested using TrypLE™ Select (Gibco) for 20 min at 37 °C, and then MDMs were immunolabeled with anti-human CD11b directly conjugated with PE-Cy7 (clone ICRF44; Thermo Fisher Scientific). Then, cells were fixed with 4% (w/v) paraformaldehyde, permeabilized and stained for 20 min on ice with anti-SARS-CoV N protein (Rockland, Philadelphia, Pn, USA) diluted in 0.3% (w/v) saponin (PanReac AppliChem, Darmstadt, Germany) in PBS [11]. After washing, the cells were incubated for 10 min with anti-rabbit IgG Alexa Fluor 488 (Thermo Fisher Scientific) and subsequently analyzed using flow cytometry (FCM; FACSCanto II, Becton Dickinson, Basel, Switzerland). FlowJo V.9.1 software (Treestar, Inc., Ashland, OR, USA) was used for analysis of phagocytosis and viral infection of the cells. After exclusion of doublets, cell lines were identified as CD11b^−^/CTV^+^; MDM as CD11b^+^; the percentage of phagocytosis was defined as the percentage of CTV^+^ MDM (Supplementary Fig. S1).

For confocal microscopy analyses, sorted CD14^+^ monocytes were labeled for 30 s with PKH26 Red Fluorescent Cell Linker (Sigma‒Aldrich) following the manufacturer’s instructions. Then, the monocytes were seeded in 8-chamber Lab-Tek II slides (Nunc, Thermo Fisher Scientific) previously coated with poly-D-lysine hydrobromide (Sigma‒Aldrich) for differentiation into MDMs as described above. SARS-CoV-2-infected cell lines were added to MDMs for 2 h. After washing nonadherent cells (as described above), the cells were cocultured with MDMs for 18 h in MDM medium. After fixation of cells with 4% (w/v) paraformaldehyde, SARS-CoV-2 nucleocapsid (NC) protein was immunolabeled as previously described, and slides were mounted with Mowiol® (Sigma‒Aldrich). Images were acquired at a resolution of 1024 × 1024 pixels using a Nikon confocal A1 microscope combined with an Eclipse Ti microscope (Nikon AG, Egg, Switzerland) and NIS-Elements AR software (v.3.30.02). Z-stack images were obtained with steps of 0.2 μm over a thickness of 10 μm using the ×20 objective with sequential and not simultaneous channel acquisition. The acquisition of images was performed with an optimized voxel size and an automatic threshold to provide high-resolution images. Generated images were analyzed using Imaris (v.8.0.2) software (Bitplane AG, Zurich, Switzerland). To avoid false-positive emissions, different settings were applied, including background subtraction, threshold applications, gamma correction, and maxima; specific conditions for each channel were applied with the same values to every image.

### Cytokine analysis

The levels of human proinflammatory cytokines, including IFN alpha 2a (IFN-α), IL-6, and TNF, were determined from collected cell culture supernatants using commercial ELISA kits from R&D Systems (Abingdon, UK,) in addition to the experiments evaluating the effect of macrophage phagocytosis of SARS-CoV-2-infected Vero E6 cells on IFN-β and IL-1β levels. The detection limits were 3.13 pg/ml for IFN-α, 9 pg/ml for IL-6, 30 pg/ml for TNF, 10 pg/ml for IFN-β, and 4 pg/ml for IL-1β.

### Statistics

GraphPad Prism 8 Software (GraphPad Software, Inc., La Jolla, San Diego, CA, USA) was used for the generation of figures and data analyses in the current study. Every experiment was independently performed at least three times with cells from different donors in triplicate if not otherwise indicated. The group differences in the percentages (infected cells or phagocytosis) and levels of cytokine expression were assessed by one- or two-way ANOVA with a post hoc Tukey correction for multiple comparisons for data organized in columns or grouped, respectively. *p* values lower than 0.05 were considered statistically significant. In the figures, the following labels were used: *p < 0.05, **p ≤ 0.002, ***p ≤ 0.001 and ****p ≤ 0.0001. Correlation analyses used Spearman’s rho analysis and were considered relevant with *R*^2^ > 0.5 (or <−0.5) and *p* < 0.05.

## Results

### SARS-CoV-2 nucleocapsid is detected in MDMs upon phagocytosis of infected cells

**Fig. 1:**
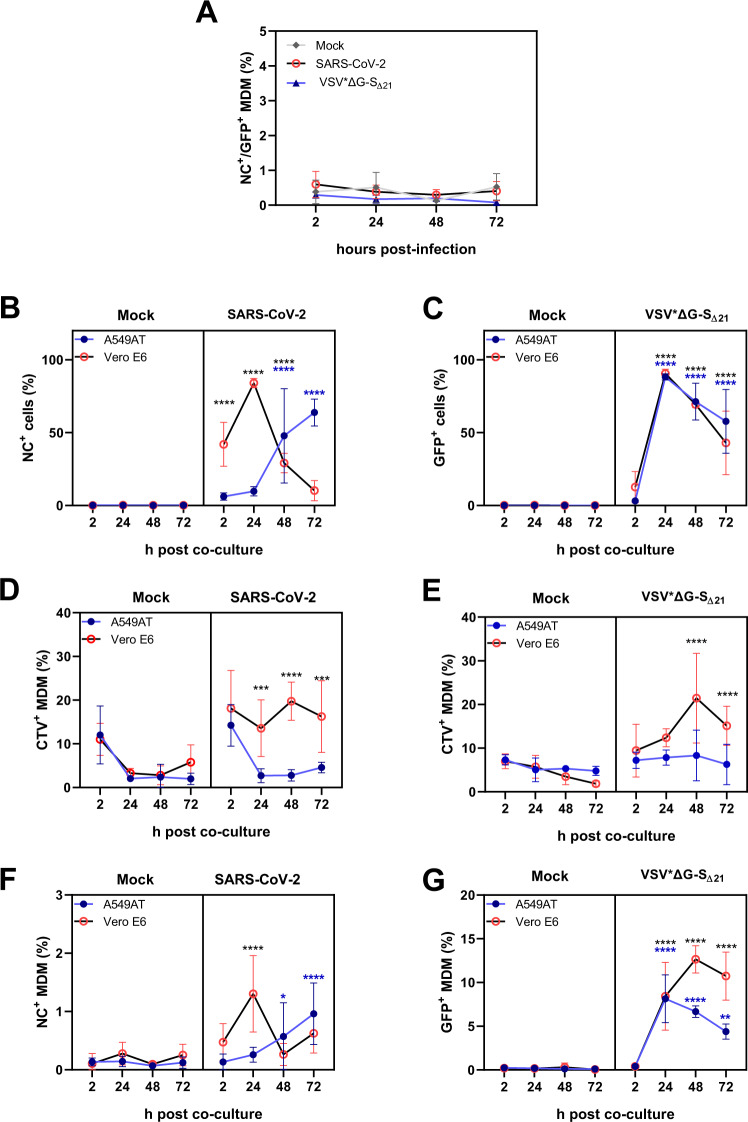
Viral protein detection in MDMs following phagocytosis of infected cells. **A** NC or GFP expression of MDMs incubated with SARS-CoV-2 (red) or pseudovirus (VSV*ΔG-S_Δ21_, in blue) and analyzed after 2, 24, 48 or 72 h by FCM. For B-G, Vero E6 or A549AT cells were infected with SARS-CoV-2 or VSV*ΔG-S_Δ21_. After 18 h, Vero E6 and A549AT cells were labeled with CTV and incubated with MDM for another 2 h. After washing the cells three times to remove nonadherent cells, the cultures were incubated for another 2–72 h and analyzed by FCM to determine the percentage of NC^+^/GFP^+^ cells and CTV^+^ macrophages at 2, 24, 48 and 72 h post coculture. **B** NC expression in Vero E6 and A549AT cells infected with SARS-CoV-2. **C** GFP expression in Vero E6 and A549AT cells infected with VSV*ΔG-S_Δ21_. **D** Frequency of CTV^+^ MDMs following phagocytosis of Vero E6 or A549AT cells (both mock-treated or SARS-CoV-2-infected). **E** Frequency of CTV^+^ MDMs following phagocytosis of Vero E6 or A549AT cells (mock-treated VSV*ΔG-S_Δ21_-infected). **F** Frequency of NC^+^ MDMs after coculture with SARS-CoV-2-infected Vero E6 or A549AT cells. **G** Frequency of GFP^+^ MDMs after coculture with VSV*ΔG-S_Δ21_-infected Vero E6 or A549AT cells. The data are from three independent experiments run in triplicate (Vero E6 cells) or duplicate (A549AT cells and VSV*ΔG-S_Δ21_ in **A**) and are presented as the mean ± standard deviation. **A**–**G** Statistically significant differences between virus-treated cells and mock-treated cells are marked by black or blue asterisks for Vero E6 or A549AT cells, respectively (*p < 0.05, **p ≤ 0.002, ***p ≤ 0.001 and ****p ≤ 0.0001)

We previously described that SARS-CoV-2 cannot infect MDMs [11]. Considering that macrophages stained positive for viral antigen in the lymph nodes of COVID-19 patients [10], we tested whether this positive staining could have resulted from the phagocytosis of SARS-CoV-2-infected cells. As shown in Fig. 1A, neither SARS-CoV-2 nor the VSV*ΔG-S_Δ21_ surrogate virus were able to infect MDM in terms of NC or GFP expression up to 72 h post infection (hpi), confirming our previous observations. We next tested whether it would be possible to detect the SARS-CoV-2 NC antigen in MDMs following phagocytosis of infected cells by flow cytometry (FCM). Supplementary Fig. S1 shows a representative set of data demonstrating phagocytosis of infected Vero E6 cells detected as CTV expressing CD11b^+^ MDMs (right panels in the second and third rows). Some of these cells also showed positive staining for NC (*y*-axis, left panels in the second and third rows). Notably, NC^+^ MDMs were always also CTV^+^, indicating that phagocytosis of infected cells was required for NC detection (data available on request).

As expected, both Vero E6 and A549AT cells in these cocultures with MDM were susceptible to infection by SARS-CoV-2, but the kinetics of infection were faster in Vero E6 than in A549AT cells and reached a maximum of infected cells at 48 hpi (corresponding to 24 h post coculture; Fig. 1B). In contrast, VSV*ΔG-S_Δ21_, a chimeric vesicular stomatitis virus (VSV) encoding the SARS-CoV-2 spike protein in place of the VSV G protein, replicated in both cell lines with similar kinetics (Fig. 1C).

Figure 1D, E summarizes the frequency of phagocytosed Vero E6 and A549AT cells by MDMs, demonstrating that the percentage of CTV^+^ MDMs was higher when exposed to infected cells compared to mock-treated cells. We also observed increased phagocytosis of infected Vero E6 cells compared to infected A549AT cells (Fig. 1F, G). The kinetics of NC/GFP expression by the infected cells (Fig. 1B, C) mirrored the detection of NC/GFP in MDMs (Fig. 1F, G), indicating that the presence of NC in MDMs is the result of phagocytosis of SARS-CoV-2-infected cells. This finding was supported by the positive correlations found between virus-positive cell lines and virus-positive MDMs following coculture with VSV*ΔG-S_Δ21_-infected A549AT cells (*R*^2^ = 0.789, *p* = 4.701e^−6^), VSV*ΔG-S_Δ21_-infected Vero E6 cells (*R*^2^ = 0.522, *p* = 0.009) and SARS-CoV-2-infected A549AT cells (*R*^2^ = 0.760, *p* = 7.351e^−8^). For MDMs cocultured with SARS-CoV-2-infected Vero E6 cells, the correlation was weak (*R*^2^ = 0.33, *p* = 0.048). Nevertheless, when removing the data from 72 h post coculture, a strong correlation between these two parameters (*R*^2^ = 0.674, *p* = 1.68e^−4^) was found. This observation could be related to the strong cytopathic effect induced by SARS-CoV-2 on Vero E6 cells at 72 h (Fig. 1B).

**Fig. 2:**
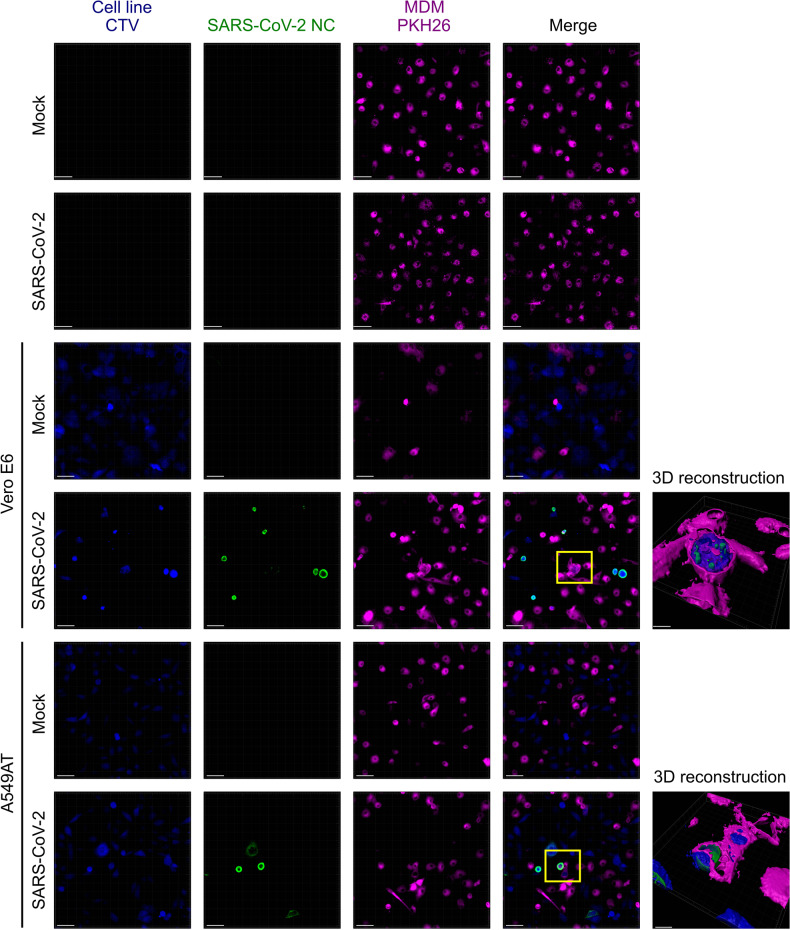
Visualization of SARS-CoV-2 NC in MDMs after phagocytosis of infected cells. Monocytes were labeled with PKH26 (violet) and differentiated into MDMs, which were cocultured for 2 h with SARS-CoV-2-infected cell lines that were labeled with CTV (blue). After washing off nonadherent cells, the coculture system was incubated for 18 h and stained for viral NC (green). Scale bar in the main panels represents a length of 40 µm, and 10 µm for the 3D reconstruction of the cells from the magnification areas in yellow. SARS-CoV-2 was observed only in MDMs after phagocytosis of infected cells, as highlighted in the 3D reconstruction magnification boxes (for Vero E6 and A549AT cells)

We also studied phagocytosis by confocal microscopy. PKH-26®-stained (purple) MDMs were cocultured for 2 h with SARS-CoV-2-infected Vero E6 or A549AT cells that had been labeled with CTV (blue), followed by two wash steps and overnight incubation and NC labeling (green). For both Vero E6 and A549AT cells, we confirmed the simultaneous presence of both SARS-CoV-2 antigen (NC^+^) and phagocytosed cell components (CTV^+^) in MDMs (Fig. 2). 3D reconstructed images demonstrate the presence of NC within CTV^+^ cellular structures inside MDMs. In conclusion, our data demonstrate that MDMs acquired the SARS-CoV-2 antigen by phagocytosis of infected cells rather than by direct infection.

For simplification, MDMs that have phagocytosed cell lines will now be termed “MDM-φ-VeroE6” (or “MDM-φ-A549AT”) for the mock controls and “MDM-φ-VeroE6^inf^” (or “MDM-φ-A549AT^inf^”) when infected cell lines were used.

### Increased IL-6 and TNF secretion by MDMs after phagocytosis of SARS-CoV-2-infected cells

**Fig. 3:**
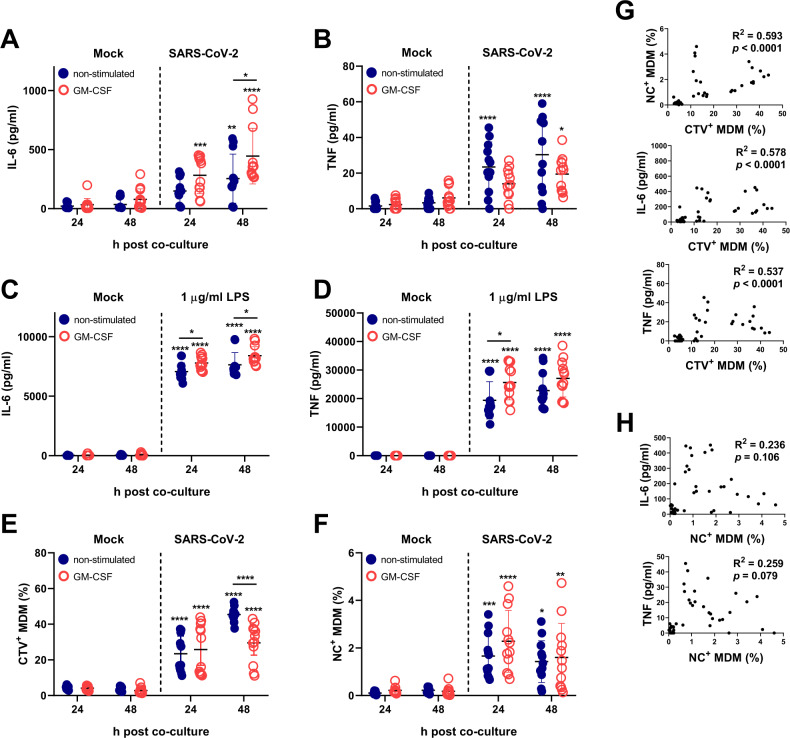
Pro-inflammatory cytokines released by MDMs after phagocytosis of SARS-CoV-2-infected cells. MDMs were either stimulated or not with human GM-CSF for 18 h prior to coculture with SARS-CoV-2-infected cells for 2 h. After three washes with warm PBS, the cells were cultured for 24 h or 48 h. **A**, **B** IL-6 and TNF levels induced in cocultures. **C**, **D** IL-6 and TNF levels induced by LPS. **E** CTV^+^ MDMs at the indicated time points in the cocultures. **F** NC^+^ MDMs at the indicated time points in the cocultures. **G** Correlation analyses for CTV^+^ MDM with NC^+^ MDM, CTV^+^ MDM with IL-6 and CTV^+^ MDM with TNF levels (top to bottom) at 24 h post coculture. **H** Correlation analysis for NC^+^ MDMs with IL-6 levels and NC^+^ MDMs with TNF levels at 24 h post coculture. The figures represent data from four different experiments with cells from different blood donors, and each experiment was performed in triplicate. The results are presented as scatter plots showing all points with the mean ± standard deviation (**A**–**F**). Statistically significant differences between mock or treated cells are indicated by asterisks, and the differences between non and GM-CSF-treated MDMs are indicated by asterisks on a bar (*p < 0.05, **p ≤ 0.002, ***p ≤ 0.001 and ****p ≤ 0.0001)

In our previous work, we demonstrated that SARS-CoV-2, in contrast to human coronavirus 229E, is unable to activate MDMs in terms of TNF, IFN-β, IL-6 and IL-1β release [11]. However, as demonstrated in Fig. 3, phagocytosis of infected cells results in cytokine production by macrophages. To extend our understanding of this macrophage response, we also included GM-CSF-prestimulated MDMs. Our data show that MDM-φ-VeroE6^inf^ but not MDM-φ-VeroE6 (MDMs that phagocytosed mock-treated VeroE6 cells) secreted both IL-6 and TNF (Fig. 3A, B, respectively), although the activation levels were lower compared those of LPS-activated MDMs (Fig. 3C, D). GM-CSF stimulation enhanced IL-6 but not TNF responses in MDM-φ-Vero E6^inf^ cells (Fig. 3A, B). In contrast, for LPS-activated MDMs, GM-CSF enhanced both IL-6 and TNF secretion (Fig. 3C, D). IL-1β and IFN-β were not detected in any of these cultures. Figure 3E shows a slight reduction in the phagocytosis rate of GM-CSF-stimulated MDMs at 48 h of culture (Fig. 3E), which did not affect the number of NC^+^ MDMs that reached levels of up to 5% (Fig. 3F). Interestingly, at 24 h of culture, we found a positive correlation between phagocytosis of SARS-CoV-2-infected cells and cytokine responses (Fig. 3G), supporting the idea that phagocytosis is required for macrophage activation. As expected, phagocytosis also correlated with the detection of viral NC. However, no significant correlations were found between cytokine secretion and NC detection in MDMs (Fig. 3H).

**Fig. 4:**
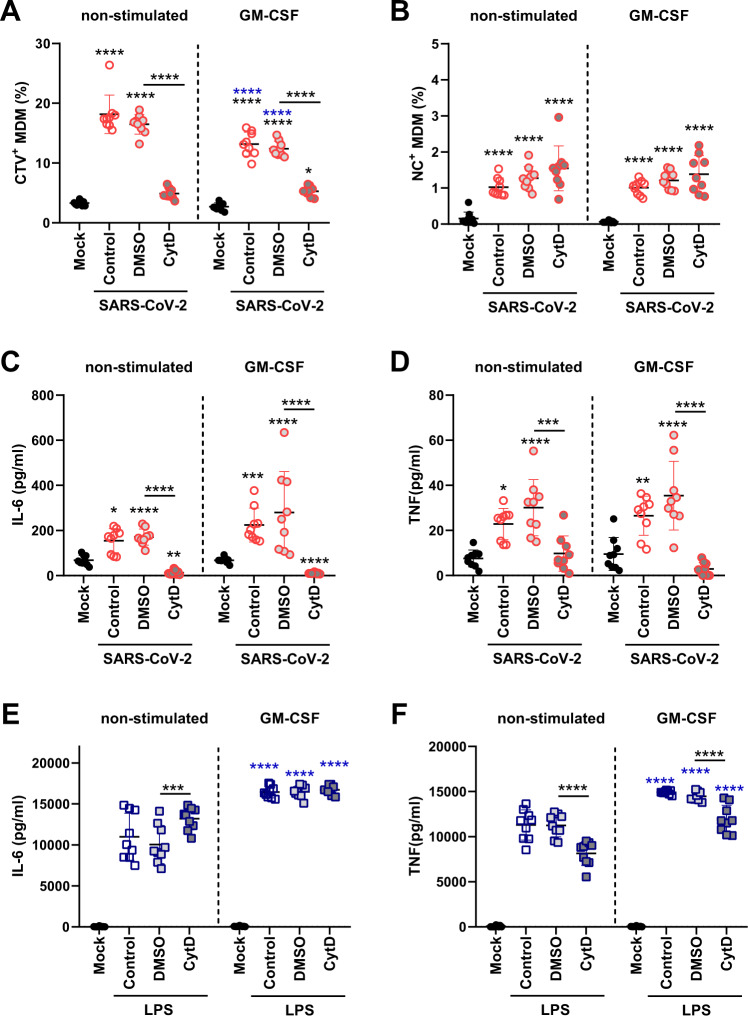
Role of phagocytosis of SARS-CoV-2-infected cells in proinflammatory cytokine secretion by MDMs. MDMs were either stimulated or not with GM-CSF for 18 h and cocultured with SARS-CoV-2-infected cells in the absence or presence of the phagocytosis inhibitor cytochalasin D (CytD) for 2 h. After washing, the cells were cocultured for 24 h in the presence or absence of CytD. DMSO was included as a control. **A**, **B** Frequencies of CTV^+^ and NC^+^ MDMs. **C**, **D** IL-6 and TNF levels in supernatants. **E**, **F** IL-6 and TNF levels induced by LPS-activated MDMs cultured for 24 h. The figures represent data from three different experiments run in triplicate. The results are presented as scatter plots showing the points and mean ± standard deviation (**A**–**F**). Statistically significant differences between mock- and GM-CSF-treated cells are indicated by asterisks (**A**–**D**), and differences between other conditions are indicated by asterisks on a bar. The differences between non and GM-CSF-treated MDMs are indicated by blue asterisks (*p < 0.05, **p ≤ 0.002, ***p ≤ 0.001 and ****p ≤ 0.0001)

Following the addition of the actin polymerization inhibitor CytD, we observed a strong decrease in the percentage of CTV^+^ MDMs after coculture with SARS-CoV-2-infected cells (Fig. 4A). Interestingly, CytD treatment did not reduce the percentage of NC^+^ MDMs (Fig. 4B). Furthermore, when the frequencies of NC^+^ MDMs within the CTV^+^ cell gate were calculated, CytD even increased the percentage of NC^+^ MDMs. The frequencies of the NC^+^ subset within CTV^+^ MDMs were 5.69% (±SD 1.39) and 7.95% (±SD 2.43) for non and GM-CSF-stimulated MDMs, respectively. Similar values were found in the DMSO-treated MDMs (7.82% and 9.89% for non and GM-CSF-stimulated MDMs, respectively). However, following treatment with CytD, the NC^+^ MDM frequencies with the CTV^+^ subset increased to 31.35% (±SD 9.87) and 27.16% (±SD 11.01) for non and GM-CSF-stimulated MDMs, respectively. This finding can be explained by the fact that CytD prevents actin polymerization to inhibit both phagocytosis and phagolysosome formation and thereby reduces the proteolytic degradation of phagocytosed proteins in the endosomal compartment [37–39]. In contrast to NC, CTV has been designed as a highly stable cellular dye.

Importantly, the secretion of IL-6 (Fig. 4C) and TNF (Fig. 4D) by MDM-φ-VeroE6^inf^ was abrogated by the phagocytosis inhibitor. In contrast, CytD did not inhibit LPS-induced IL-6 production (Fig. 4E) and only partially reduced LPS-induced TNF production (Fig. 4F). The latter result is in accordance with the results of a previous work [40].

### After phagocytosis of virus-infected cells, MDMs induce strong IFN-α production by pDC activation

**Fig. 5:**
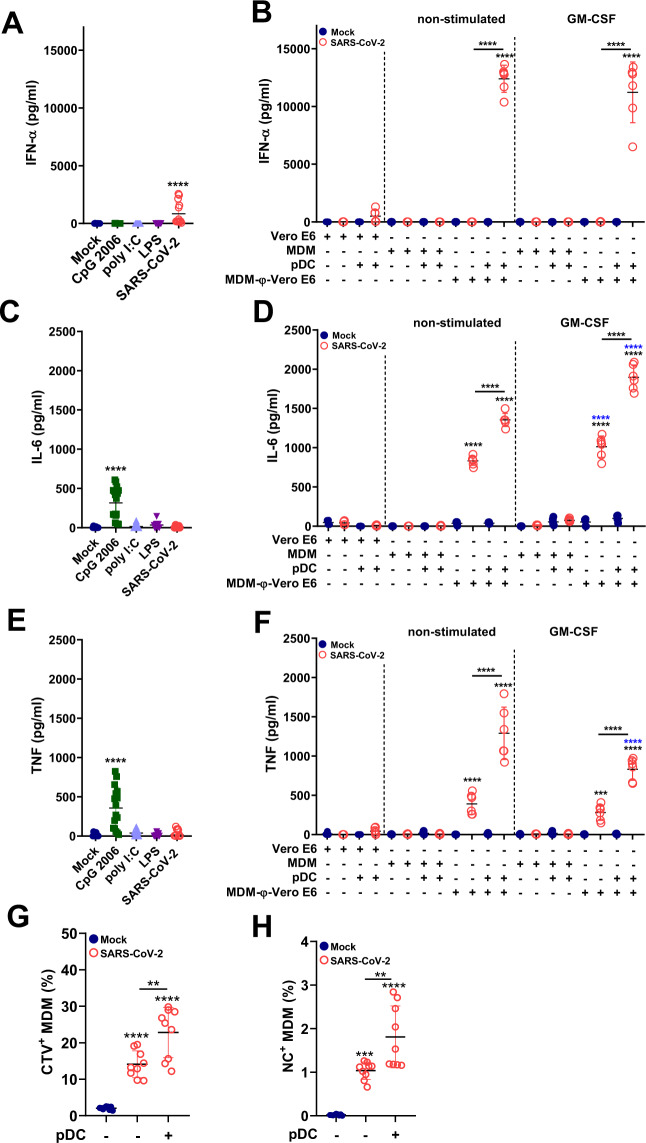
IFN-α and proinflammatory cytokine production in cocultures of pDCs with MDM-φ-VeroE6^*inf*^. MDMs were either stimulated or not with GM-CSF for 18 h prior to coculture with SARS-CoV-2-infected cells for 2 h (MDM-φ-VeroE6^*inf*^). After three washes, pDCs were added for 18 h to the cultures, and the supernatants were tested for cytokines. Controls for the different cell types cultured alone were included. **A** IFN-α responses of pDCs cultured alone and stimulated by different ligands or SARS-CoV-2 (MOI of 1 TCID_50_/cell). **B** IFN-α responses in the cocultures as indicated on the *x*-axis. **C** IL-6 responses of pDCs alone stimulated as in (**A**). **D** IL-6 responses in the cocultures. **E** TNF responses of pDCs alone stimulated as in (**A**). **F** TNF responses in the cocultures. **G**, **H** Frequency of CTV^+^ and NC^+^ MDM-φ-VeroE6^*inf*^ cultured in the presence or absence of pDCs for 18 h of culture. The *x*-axes in (**B**), (**D**), and (**F**) indicate the cell types added to the cultures. “MDM-φ-VeroE6”: MDM that had phagocytosed Vero E6 cells. **A**, **C**, **E** represent data from cells of 4 to 9 different blood donors run in duplicate (single replicate for one donor) in independent experiments. **B**, **D**, **F**–**H** represent data from 6 to 9 different donors run in single replicates in independent experiments. The results are presented as scatter plots indicating the mean ± standard deviation. Statistically significant differences in the values compared to mock-treated cells are indicated by black asterisks; differences between untreated and GM-CSF-treated MDMs are indicated with blue asterisks; statistically significant differences for the absence or presence of pDCs are indicated by asterisks on a bar (*p < 0.05, **p ≤ 0.002, ***p ≤ 0.001 and ****p ≤ 0.0001)

MDM are poor producers of IFN-I; in fact, as mentioned above, we did not detect any secretion of IFN-β by MDM-φ-VeroE6^inf^. Given the extraordinary ability of pDCs to sense virus-infected cells, resulting in potent IFN-α secretion [31, 33, 34], we added pDCs to MDM-φ-VeroE6^inf^ and measured the levels of the released cytokines in the cell culture supernatants. As previously described [20–24], pDCs produced IFN-α following direct exposure to SARS-CoV-2 (Fig. 5A). We noted a high variability in this response among the different tested donors, of which 64% were classified as low responders (51–361 pg/ml of IFN-α) and 36% as high responders (1384–2571 pg/ml of IFN-α). The coculture of pDCs with SARS-CoV-2-infected Vero E6 cells resulted in similar levels of IFN-α secretion (Fig. 5D, 40–1309 pg/ml). However, when pDCs were cocultured with MDM-φ-VeroE6^inf,^ a much higher IFN-α response was observed in all tested blood donors (6531–13,650 pg/ml; Fig. 5B).

Direct stimulation of pDCs by SARS-CoV-2 or SARS-CoV-2-infected Vero E6 cells did not induce significant IL-6 secretion (Fig. 5C, D). However, coculture of pDCs with MDM-φ-VeroE6^inf^ enhanced IL-6 levels compared to those of MDM-φ-VeroE6^inf^ cultured alone. This pDC-dependent enhancement was found in both nonstimulated and GM-CSF-stimulated MDMs (Fig. 5D). A similar observation was made for TNF, which was not significantly induced by direct stimulation with SARS-CoV-2 or with virus-infected Vero E6 cells (Fig. 5E, F). However, coculture of pDCs with MDM-φ-VeroE6^inf^ resulted in a strongly enhanced TNF production compared to that of MDM-φ-VeroE6^inf^ alone (Fig. 5F).

Comparable findings were made following coculture of pDCs with MDM-φ-A549AT^inf cells^ (Supplementary Fig. S2), indicating that the potent pDC activation mediated by macrophages that had phagocytosed virus-infected cells was not dependent on the cell line type used. No increased cytokine responses were found when pDCs were in contact with either MDM-φ-VeroE6 or MDM-φ-A549AT (Figs. 5B, D, F and S2, respectively).

Interestingly, the addition of pDCs to the coculture system resulted in a significant increase in MDM phagocytosis rate of SARS-CoV-2-infected cells (Fig. 5G), which also increased the frequency of NC^+^ MDMs (Fig. 5H). Altogether, these results demonstrate that pDCs can efficiently sense macrophages that have phagocytosed SARS-CoV-2-infected cells, independent of the cell type, resulting in extraordinarily strong IFN-I secretion, which in turn enhances the phagocytosis of infected cells and proinflammatory cytokine responses in cocultures. These results suggest an intercellular communication axis between macrophages and pDCs during antiviral responses.

### Role of IFN-α in MDM functional modulation

**Fig. 6:**
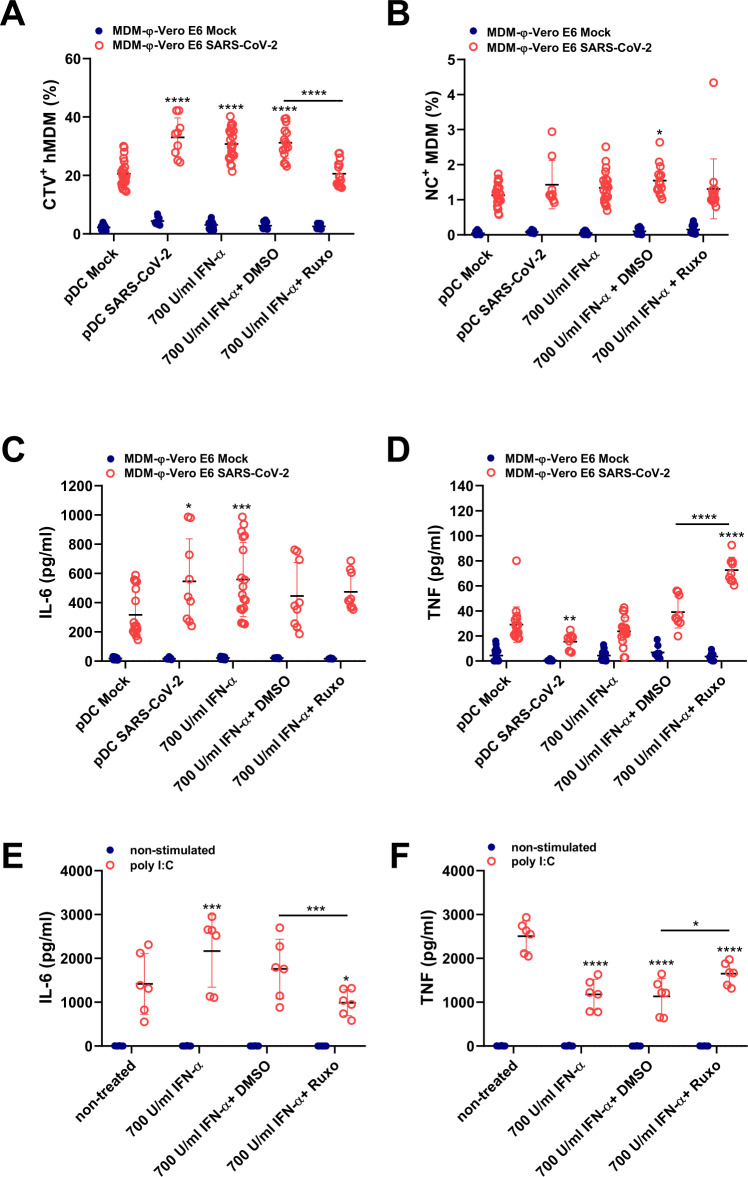
Functional modulation of MDMs by pDC-conditioned medium or IFN-α. MDMs were either stimulated with supernatants from pDCs previously activated with SARS-CoV-2 (MOI of 1 TCID_50_/cell; “pDC SARS-CoV-2”) or with IFN-α2b at 700 U/ml. For the inhibition of the IFN-I pathway, MDMs were treated with ruxolitinib (Ruxo) at 10 µg/ml. DMSO at 0.1% v/v was included as a negative control. Supernatants from nonstimulated pDCs (“pDC Mock”) were used as negative controls. After 24 h of MDM stimulation, mock-treated or SARS-CoV-2-infected Vero E6 cells were added to the MDMs for a total of 24 h in coculture (**A**–**D**). **A** Frequency of CTV^+^ MDMs. **B** Frequency of NC^+^ MDMs. **C**, **D** IL-6 and TNF levels in the supernatants. **E**, **F** IL-6 and TNF levels in poly I:C (10 μg/ml)-activated MDM cultures. The results represent scatter plots showing all points with mean ± standard deviation from three different experiments run in triplicate for the cocultures and in duplicate for the poly I:C controls. Statistically significant differences of treated samples compared to the negative control group (“pDC Mock” or “nontreated”) are indicated with asterisks. Differences between other groups are indicated with asterisks on a bar. (*p < 0.05, **p ≤ 0.002, ***p ≤ 0.001 and ****p ≤ 0.0001)

The pDC-mediated enhancement of MDM phagocytosis and secretion of IL-6 and TNF in the cocultures motivated us to investigate this phenomenon in more detail. We tested whether the treatment of MDMs with IFN-α during coculture with infected cells had a similar effect on the phagocytosis of cells and secretion of the proinflammatory cytokines IL-6 and TNF as conditioned medium from SARS-CoV2-activated pDCs. MDMs were stimulated with supernatant from SARS-CoV-2-stimulated pDCs or with recombinant human IFN-α for 24 h, followed by the addition of SARS-CoV-2-infected cells for another 24 h. As shown in Fig. 6A, IFN-α enhanced phagocytosis in a comparable manner to pDC supernatants. Treatment of MDMs with a JAK1/2 inhibitor (ruxolitinib) prevented the effect of IFN-α on MDM phagocytosis (Fig. 6A), although this was not reflected in the frequency of NC^+^ MDMs (Fig. 6B). MDM-φ-VeroE6^inf^ exposed to IFN-α or pDC supernatants produced higher levels of IL-6 (1.72- and 1.76-fold increase, respectively). This increase was similar to the increase caused by coculture with pDCs (1.63-fold increase; Figs. 5D and 6C), indicating that the enhanced IL-6 levels induced in pDC-MDM-φ-VeroE6^inf^ cocultures (Fig. 5D) are at least partially of macrophage origin. Ruxolitinib reversed the IFN-α-mediated enhancement of IL-6 production in poly I:C-stimulated cells (Fig. 6E) but failed to significantly inhibit the effect of IFN-α on MDM-φ-VeroE6^inf^ cells.

Interestingly, IFN-α appeared to have opposite effects on TNF production. pDC supernatants decreased TNF production in MDM-φ-VeroE6^inf cells^ (Fig. 6D). Nevertheless, due to the low levels of TNF in MDM-φ-VeroE6^inf cells^, it was difficult to assess the impact of IFN-α on this mode of macrophage activation. For these reasons, we evaluated poly I:C-induced TNF responses in MDMs, in which an inhibitory effect of IFN-α was confirmed (Fig. 6F). This inhibitory effect of IFN-α was also supported by the observation that ruxolitinib-treated MDM-φ-VeroE6^inf^ cells produced significantly higher levels of TNF than those produced by the DMSO controls (Fig. 6D).

Overall, these results indicated that the increase in IL-6 levels in cocultures of pDCs with MDM-φ-VeroE6^inf^ compared to MDM-φ-VeroE6^inf^ alone is caused at least in part by the enhancing effect of IFN-α on MDMs. In contrast, for the TNF responses, our data indicate that the enhanced TNF found in pDC-MDM-φ-VeroE6^inf^ cocultures is pDC-derived.

### Requirements of cognate interactions between pDCs and macrophages

**Fig. 7:**
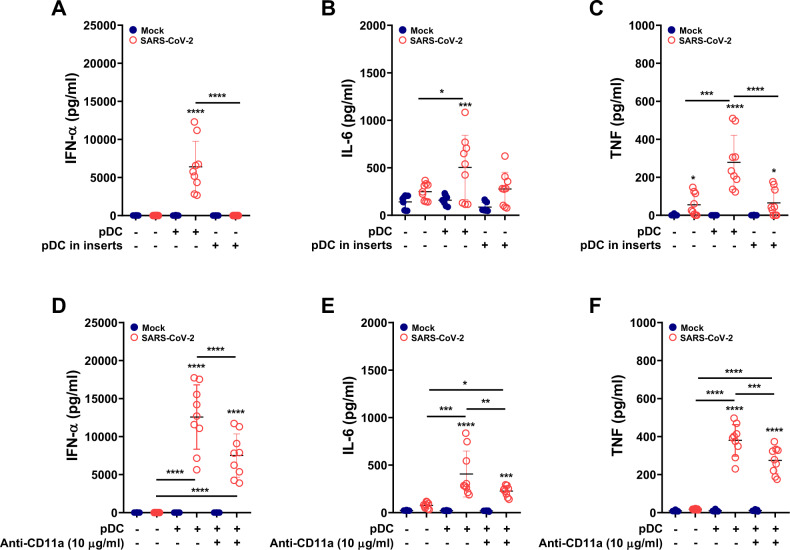
Requirement of cell contact for pDC sensing of MDM-φ-VeroE6^*inf*^. MDMs were cocultured with SARS-CoV-2-infected cells for 2 h, washed three times and cocultured with pDCs for a total of 18 h. In (**A**)–(**C**), MDMs and pDCs were either in direct coculture or physically separated by adding pDCs to inserts as indicated. In (**D**)–(**F**), the physical interaction of MDMs with pDCs was inhibited with an anti-CD11a antibody (10 μg/ml) where indicated. **A**, **D** IFN-α levels in supernatants. **B**, **E** IL-6 levels. **C**, **F** TNF levels. All experiments were run in triplicate from three independent experiments performed with cells from different donors. The results are presented as scatter plots showing all the points and the mean ± standard deviation. Statistically significant differences in the cytokine production levels compared to mock-treated cells are indicated by black asterisks; other significant differences are indicated with asterisks over a bar between the values (**p ≤ 0.002, ***p ≤ 0.001 and ****p ≤ 0.0001)

It has been previously demonstrated with various viruses that pDC sensing of infected cells requires cognate interactions with the infected cells [31, 33, 34]. As shown in Fig. 7A, physical separation of pDCs from MDMs completely abrogated the IFN-α response induced by MDM-φ-VeroE6^inf^. Moreover, the pDC-dependent increase in IL-6 and TNF levels was observed only when pDCs were in direct contact with MDMs (Fig. 7B, C), further supporting an additive/promotive role of pDCs in this cellular response to infection.

Based on previous studies demonstrating integrin-dependent tight interactions between pDCs and infected cells [31, 33], we tested the inhibitory effect of a CD11a-specific monoclonal antibody. Using this approach, a significant reduction in IFN-α, IL-6 and TNF production by pDCs stimulated with MDM-φ-VeroE6^inf^ was observed (IFN-α, IL-6 and TNF, Fig. 7D, E, respectively). Altogether, these results indicate that pDC sensing of infection in this model requires cognate interaction with MDMs.

### TLR7 and STING pathways are involved in pDC activation by MDM-φ-VeroE6^inf^

**Fig. 8:**
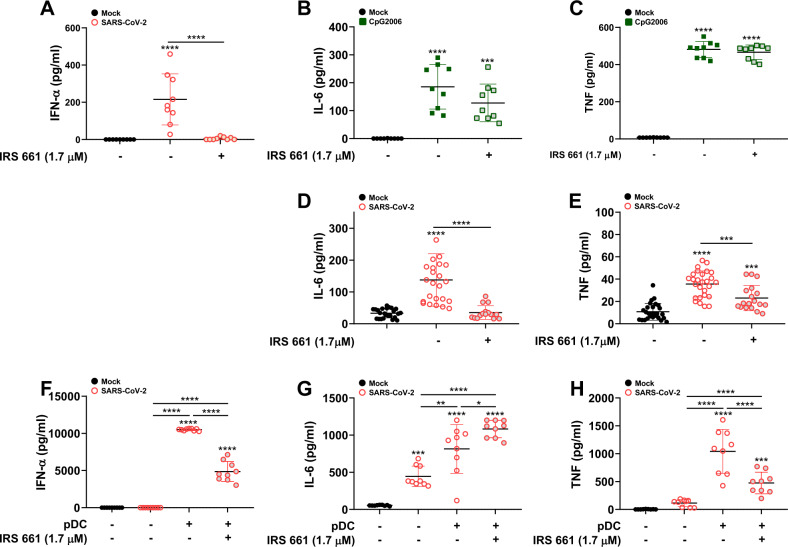
Role of TLR7 in MDM and pDC activation. TLR7-mediated sensing was inhibited with IRS661 (1.7 µM). All stimulations were performed for 18 h. **A** IFN-α levels in pDC cultures stimulated with SARS-CoV-2 supernatants. **B**, **C** IL-6 and TNF levels in pDC cultures stimulated with CpG2006. **D**, **E** IL-6 and TNF levels in MDM-φ-VeroE6^*inf*^ (MDMs that have phagocytosed infected cells). **F**–**H** IFN-α, IL-6 and TNF levels in MDM-φ-VeroE6^*inf*^ cocultured with pDCs. **A**–**C** represent data from nine different blood donors run in single replicates in independent experiments. **D**, **E** represent data from cells of 5–9 different donors run in triplicate in independent experiments. **F**–**H** represent data of cells from 8 to 9 different donors run in single replicates from independent experiments. The results are presented as scatter plots with bars showing the mean ± standard deviation. Statistically significant differences in the cytokine levels between mock and treated cells are indicated by asterisks; differences between other conditions are indicated by asterisks over a bar (*p < 0.05, **p ≤ 0.002, ***p ≤ 0.001 and ****p ≤ 0.0001)

Considering the established role of TLR7 in pDC responses to SARS-CoV-2 [25], we investigated the involvement of this endosomal sensor of single-stranded RNA in the response of both MDM-φ-VeroE6^inf^ and pDCs cocultured with MDM-φ-VeroE6^inf^. As expected, the TLR7 inhibitor IRS 661 [41] abrogated IFN-α secretion in pDCs directly exposed to SARS-CoV-2 (Fig. 8A). In line with its specificity for TLR7, IRS 661 did not inhibit IL-6 and TNF responses that were induced by the TLR9 ligand CpG2006 (Fig. 8B, C, respectively). Interestingly, IRS 661 reduced the levels of IL-6 and TNF secretion by MDM-φ-VeroE6^inf^ (Fig. 8D, E).

Importantly, IRS661 treatment of pDCs cocultured with MDM-φ-VeroE6^inf^ caused a significant reduction in IFN-α (Fig. 8F) and TNF (Fig. 8H) production but had no or even an enhancing effect on IL-6 responses (Fig. 8G). These results suggest that TLR7 plays an important but not exclusive role in pDC and MDM immune responses to SARS-CoV-2.

**Fig. 9:**
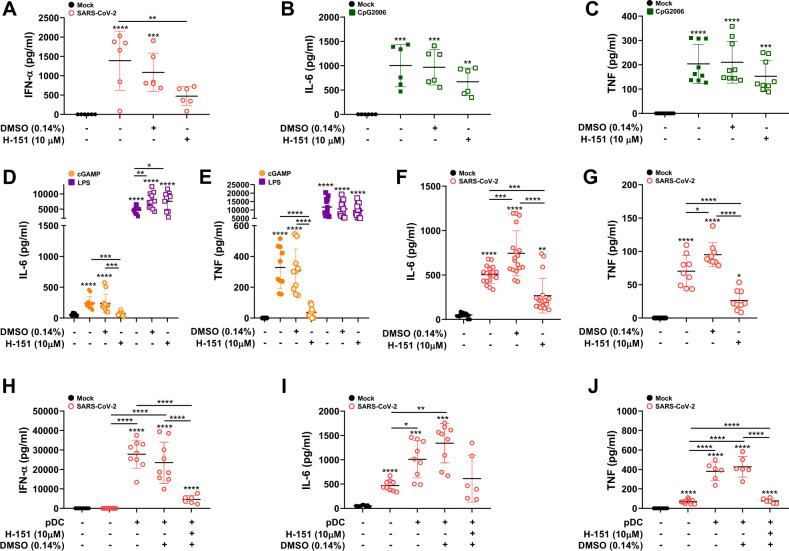
Role of the STING pathway in MDM and pDC activation. STING signaling was inhibited using the inhibitor H-151 (10 µM). The duration of stimulation for all cultures was 18 h. **A** IFN-α levels in pDC cultures stimulated with SARS-CoV-2 supernatants. **B**, **C** IL-6 and TNF levels in pDC cultures stimulated with CpG2006. **D**, **E** IL-6 and TNF levels in cultures of MDMs that were stimulated with cGAMP or LPS for 18 h in the absence or presence of H-151. **F**, **G** IL-6 and TNF levels in MDM-φ-VeroE6^*inf*^ cultures. **H**–**J** IFN-α, IL-6 and TNF levels in cocultures of pDCs and MDM-φ-VeroE6^*inf*^. In (**A**)–(**C**), cells from 6 to 9 different blood donors were used in single replicates. In (**D**) and (**E**), cells from 5 to 6 donors in duplicate were used. In (**F**) and (**G**), cells from 3 to 6 different donors were run in triplicates. In (**H**)–(**J**), cells from 5 to 9 different donors run in single replicates were used. The results are presented as scatter plots with bars indicating the mean ± standard deviation. Statistically significant differences in the cytokine levels between mock and treated cells are indicated by asterisks, and differences between other conditions are indicated by asterisks on a bar (*p < 0.05, **p ≤ 0.002, ***p ≤ 0.001 and ****p ≤ 0.0001)

The cyclic GMP-AMP synthase (cGAS)-stimulator of interferon genes (STING) pathway was demonstrated to stimulate IFN-I production in macrophages in response to mitochondrial DNA that is released from dying SARS-CoV-2-infected cells [42]. We therefore investigated the contribution of STING in our model of MDM and pDC activation using the STING inhibitor H-151 [43]. Surprisingly, we observed that H-151 decreased IFN-α production by pDCs that were directly activated by SARS-CoV-2 (Fig. 9A) without significantly affecting the responses to CpG2006 stimulation (Fig. 9B, C).

We next investigated the effect of STING inhibition on MDM responses. To confirm the reported specificity of H-151 as a STING inhibitor, MDMs were either stimulated with cGAMP (a specific STING ligand) or LPS either in the presence or absence of the inhibitor. Using this approach, we observed that H-151 inhibited only the STING pathway with respect to IL-6 and TNF responses (Fig. 9D, E). The inhibition of STING in MDM-φ-VeroE6^inf^ resulted in a significant reduction in both IL-6 and TNF levels (Fig. 9F, G). Finally, we evaluated the role of the STING pathway in pDC activation mediated by MDM-φ-VeroE6^inf^. H-151 treatment caused a clear decrease in the levels of all tested cytokines, with particularly strong effects on IFN-α and TNF (Fig. 9H–J). Altogether, these results indicate that both TLR7 and STING are involved in the IFN-I and proinflammatory cytokine responses induced in MDMs following phagocytosis of SARS-CoV-2-infected cells and in pDCs that are activated by such MDMs.

## Discussion

The analysis of host‒pathogen interactions in human infectious diseases requires suitable models that allow us to dissect the complexity of innate immune activation. In recent years, different animal models have been developed to study SARS-CoV-2 interactions with hosts, such as ferrets or transgenic mice expressing human ACE2, but these models are often not suitable for understanding the mechanisms of macrophage activation and cytokine storms found in patients suffering from severe COVID-19 [44]. Here, we developed a new in vitro model that allowed us to dissect the complex interaction of SARS-CoV-2-infected cells with macrophages and pDCs that leads to the secretion of IFN-α, IL-6 and TNF.

Our observation that macrophages are not susceptible to SARS-CoV-2 infection and are immunologically unresponsive [11] seemed to contradict previous work showing that macrophages in COVID-19 patients were positive for the SARS-CoV-2 NC antigen [10]. However, our in vitro experiments indicated that MDMs efficiently phagocytose infected cells, resulting in positive immunostaining of MDMs for the SARS-CoV-2 NC protein. It is likely that this mechanism also takes place in vivo, given that phagocytosis of dying cells is a central function of macrophages. Notably, macrophages isolated from COVID-19 patients were found to engulf dying cells, resulting in activation of the cGAS-STING pathway [42]. In fact, other studies have noted that macrophages phagocytose SARS-CoV-2-infected cells in vivo [45–47] and in vitro [25, 48].

Importantly, our present study demonstrates that phagocytosis of SARS-CoV-2-infected cells is not immunologically silent but can result in the production of the proinflammatory cytokines IL-6 and TNF, both of which are known to be involved in COVID-19 pathogenesis [49–52]. Our data indicate that this mode of macrophage activation can be enhanced by GM-CSF, which is known to promote a proinflammatory macrophage phenotype [53] and is proposed to play a role in inflammation during severe COVID-19 [49].

To understand the cellular mechanisms leading to systemic IFN-I production during COVID-19, we investigated the mode of pDC activation and identified a novel pathway of macrophage-mediated pDC activation that was found to be extremely potent. There are several reports from our group and other groups showing that the activation of pDCs by virus-infected cells can be much more prominent than the activation by viral particles, and this has been shown for a number of viruses [31–35, 54]. Beyond this direct interaction between virus-infected cells and pDCs, the present study identified a novel pathway of cell contact-dependent pDC activation that was induced by macrophages following the phagocytosis of infected cells. This transcellular contact also involved CD11a, which is known to play a role in the formation of cognate interactions between pDCs and virus-infected cells [31, 33]. This interaction with macrophages induced much higher IFN-α responses than those induced by pDC stimulated by virus or virus-infected cells. This novel mode of pDC activation also induced the secretion of IL-6 and TNF at levels that were significantly higher than those observed with MDMs alone following phagocytosis of infected cells.

As shown here, pDC-derived IFN-α stimulated the phagocytosis of infected cells by macrophages and thereby might contribute to the antiviral response. This effect of IFN-I on phagocytosis is in accordance with previous publications [55–57]. It is worth noting that IFN-I-stimulated macrophages upregulate sialoadhesin (CD169), which was shown to mediate an abortive SARS-CoV-2 infection of macrophages, resulting in the detection of viral RNA but not viral antigens in these cells [12]. The effect of IFN-α on macrophages also promotes proinflammatory cytokine responses [25], which we confirmed here for IL-6 but not for TNF. This observation is in line with a previously reported study showing that IFN-I induced the regulatory genes *Nfil3, Batf2, Themis2 and Chd1*, which suppress TLR4-stimulated TNF protein production, despite high levels of TNF gene expression [58].

We further evaluated the nature of the ligand‒receptor interaction leading to macrophage and pDC activation in our model using specific inhibitors of TLR7 and STING. For RNA viruses, including SARS-CoV-2, the triggering of endosomal TLR7 by single-stranded RNA represents a prominent pathway for pDC activation [25], which is also relevant in RNA virus-infected cell-mediated pDC activation [31, 34]. The present data suggest that TLR7 is also triggered in pDCs cocultured with MDM-φ-VeroE6^inf^.

Surprisingly, our data further showed that inhibition of the STING pathway strongly affected IFN-α levels upon stimulation of pDCs with both MDM-φ-VeroE6^inf^ and free SARS-CoV-2 virions. Along the same lines, macrophage responses to SARS-CoV-2-infected cells appeared to use both the TLR7 and STING pathways for proinflammatory cytokine responses. A possible explanation is the previously reported retinoic acid inducible gene-I (RIG-I) crosstalk with the STING pathway. For several virus infections, it was shown that a complex of the viral RNA with RIG-I is stabilized by the association with mitochondrial antiviral-signaling protein (MAVS), and this can mediate activation of STING, resulting in a cGAMP-independent IFN-I response [59]. Alternatively, macrophages may transfer immunostimulatory self-DNA to pDCs and trigger the cGAS-STING pathway. Such self-DNA can originate from disrupted mitochondria of phagocytosed cells [60], as previously reported for macrophages following phagocytosis of SARS-CoV-2-infected cells [42]. Another possibility to explore could be the transfer of cGAMP produced in macrophages to pDCs. This cGAMP directly activates the STING pathway in pDCs.

Tight interactions between cells of the immune system often involve interactions between αLβ2 integrin and ICAM-1 to form synapse-like structures, which appear to be required for transmission of material such as nucleic acids from virus-infected cells to pDCs [31, 32]. The data of the current manuscript indicate that similar pathways also support cognate interactions and immunostimulatory material transfer from macrophages that have phagocytosed infected cells to pDCs. For pDC interaction with infected cells, this intercellular communication could involve short-range exosomes [33, 61, 62], but tunneling nanotubes or gap junctions could also play a role [63]. Future research is needed to fully understand the pathways of transcellular interactions between macrophages or infected cells and pDCs that lead to very strong IFN-I responses.

Considering the role of IFN-I and inflammatory cytokine responses in the severity of COVID-19 [64], it is important to understand the cellular mechanisms and pathways involved in pDC activation. While early and robust IFN-I responses appear to be associated with mild disease, sustained secretion of IFN-I and proinflammatory cytokines was found to be involved in more severe COVID-19 [3, 4, 17, 20, 29]. Therefore, the contribution of pDCs to innate immune responses during COVID-19 and the mode of pDC activation in vivo should be investigated in future studies. A recent study using humanized mice as a model for chronic COVID-19 provides supporting evidence for the hypothesis of macrophage-mediated pDC activation. High levels of SARS-CoV-2 in the lungs of infected animals were associated with a significant increase in macrophage and pDC populations, coinciding with higher levels of IFN-I gene expression and interferon signaling gene transcripts [65].

Altogether, our data demonstrate a novel and very potent pDC activation pathway that links the phagocytosis of dying cells, a central function of macrophages, with antiviral and inflammatory responses. It will also be of interest to investigate the mechanism and role of this pathway of pDC and macrophage activation in other viral infections.

## Supplementary information

Sup-Fig1

Sup-Fig2

Supplementary Figures

## Data Availability

The data that supports the findings of this study have been uploaded and made available on Zenodo 10.5281/zenodo.7965645.
