# Supplementary figures and images for: Macrophage phagocytosis of SARS-CoV-2-infected cells mediates potent plasmacytoid dendritic cell activation
Source: Cell Mol Immunol. 2023 May 30;20(7):835–49. doi: 10.1038/s41423-023-01039-4 (PMC10227409; doi:10.1038/s41423-023-01039-4)

# Big cells

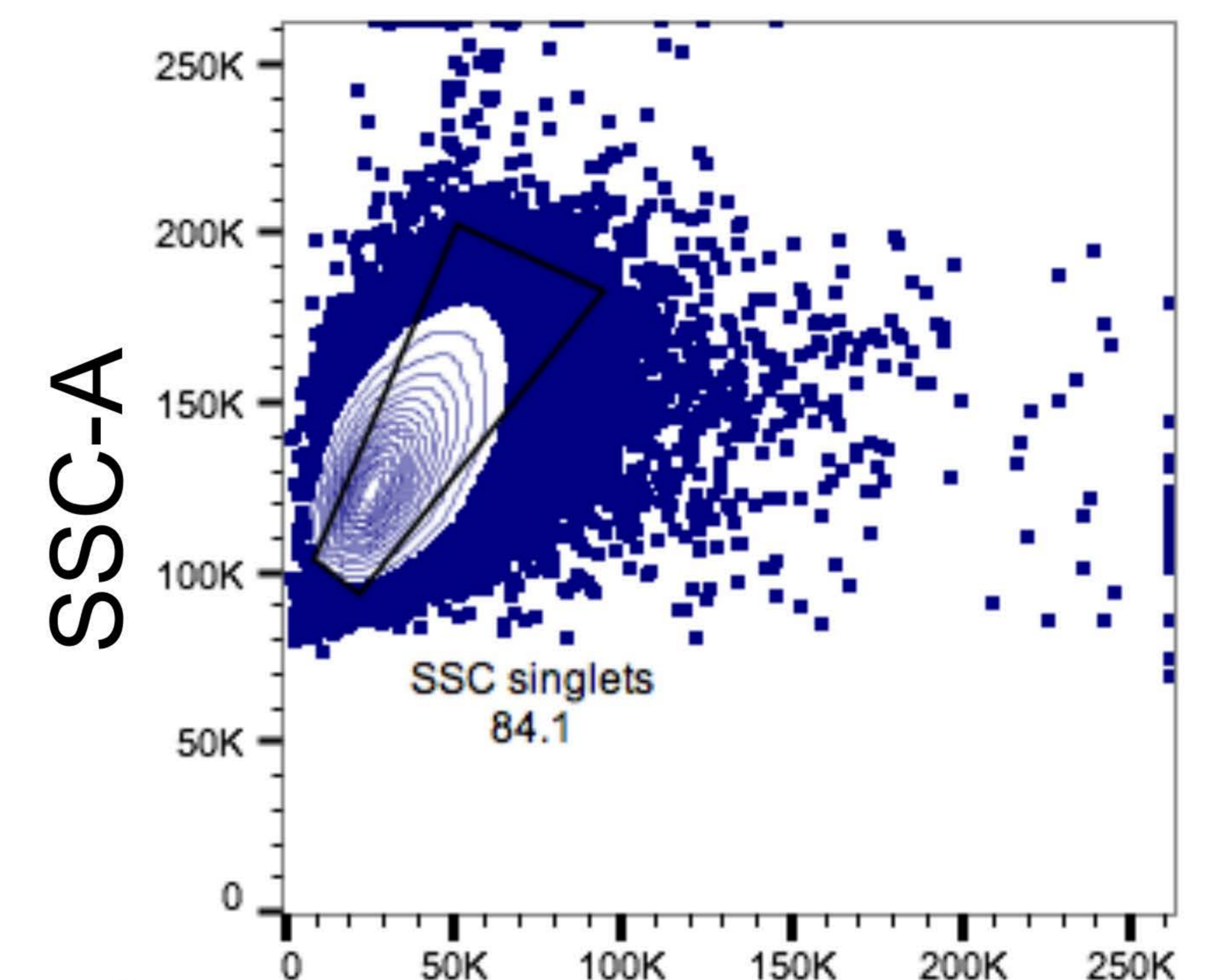

# FSC-A

MDM

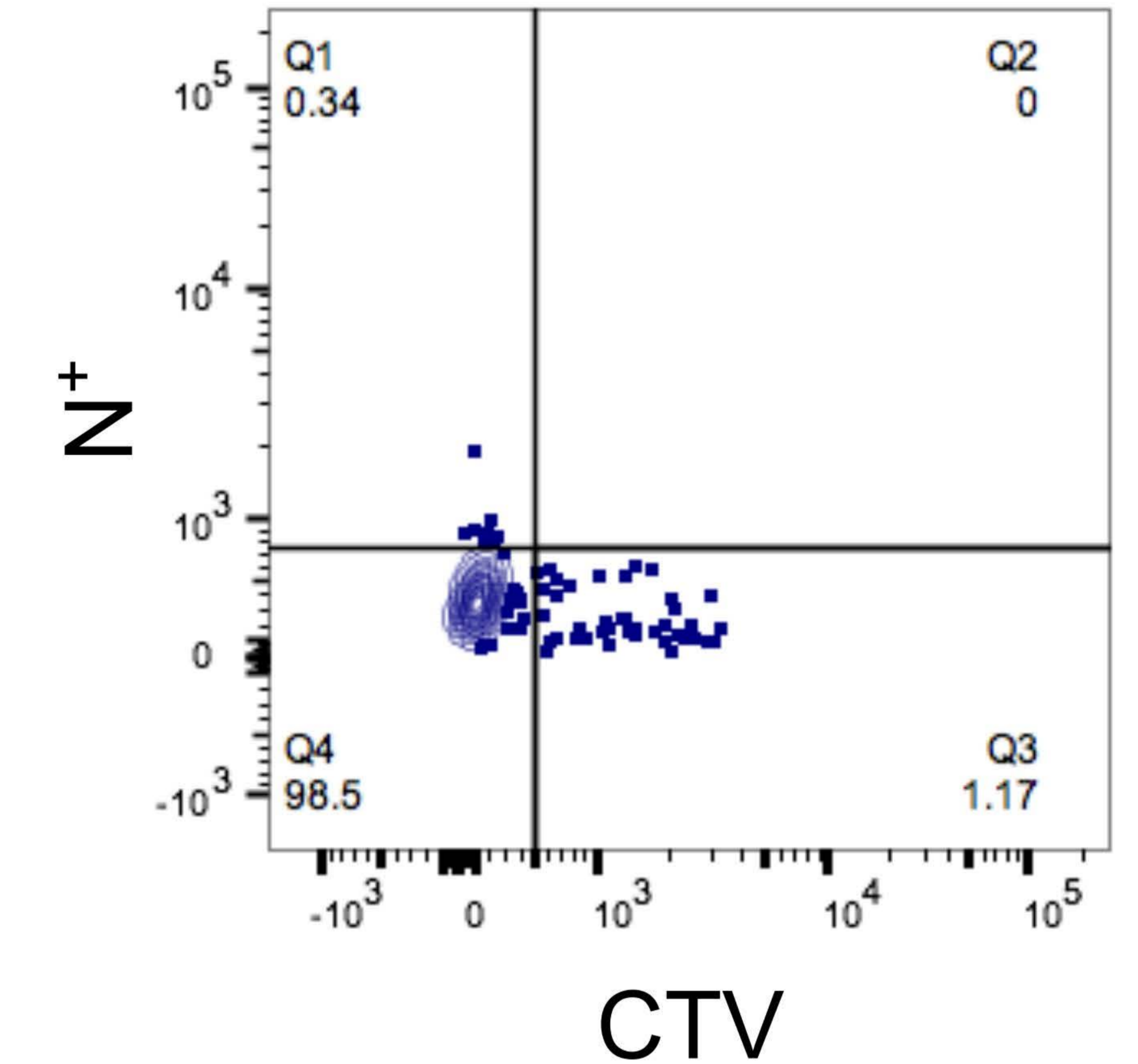

# MDM

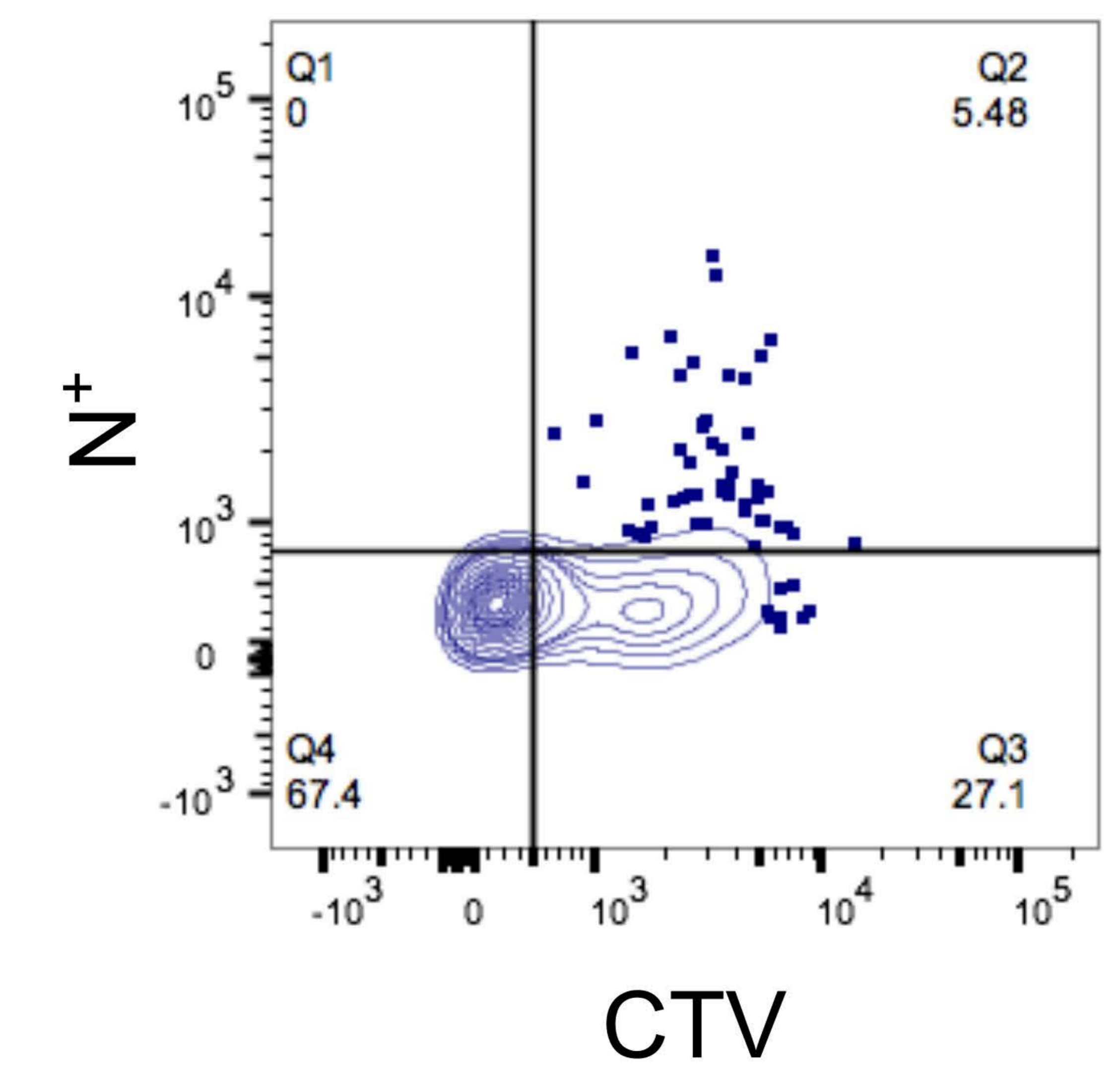

# SARS-CoV-2

Supplement: Supplementary file 1 — Sup-Fig1 [file 41423_2023_1039_MOESM1_ESM.pdf]

**A**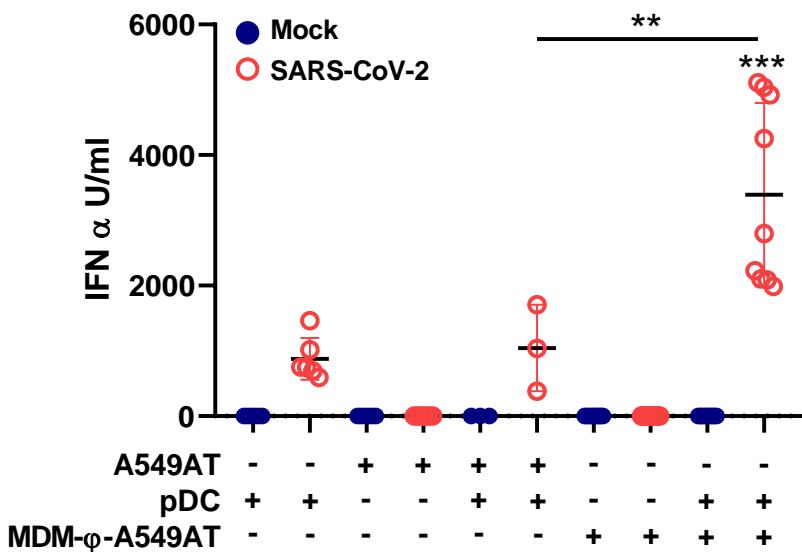**B**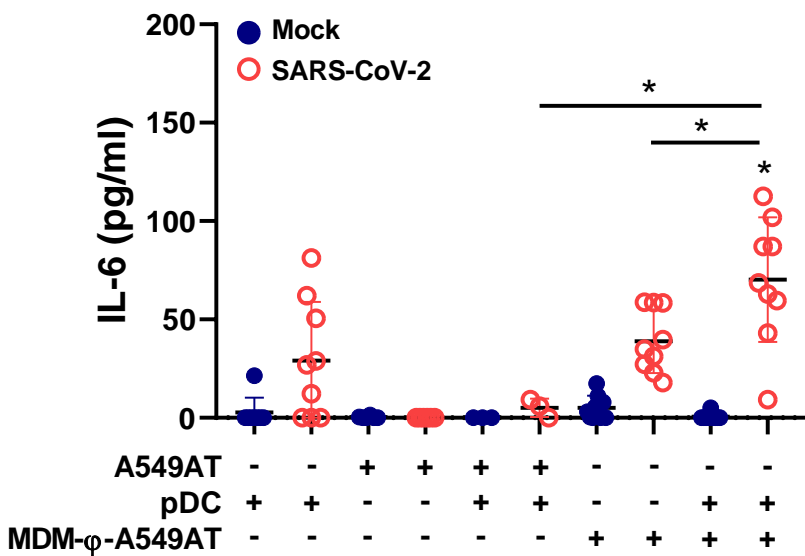**C**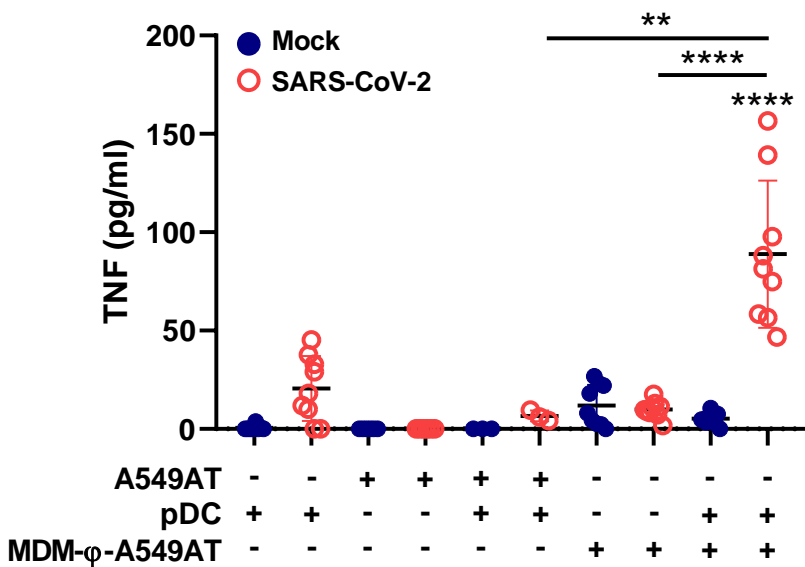

Supplement: Supplementary file 2 — Sup-Fig2 [file 41423_2023_1039_MOESM2_ESM.pdf]
