# Supplementary material for: Macrophage phagocytosis of SARS-CoV-2-infected cells mediates potent plasmacytoid dendritic cell activation
Source: Cell Mol Immunol. 2023 May 30;20(7):835–49. doi: 10.1038/s41423-023-01039-4 (PMC10227409; doi:10.1038/s41423-023-01039-4)
Supplement: Supplementary file 3 — Supplementary Figures [file 41423_2023_1039_MOESM3_ESM.docx]

**Supplementary Figure legends**

**S1. Flow cytometry gating strategy.** Vero E6 or A549AT cells were infected with SARS-CoV-2 or VSV*ΔG-S_Δ21_. After 18 h, the cells were labeled with Violet CellTrace® (CTV) and incubated with MDMs for another 2 h. After washing the cells three times to remove nonadherent cells, the cocultures were incubated for 2-72 h and analyzed by FCM at the indicated time points. The gating strategy for doublet discrimination, quantification of CTV and viral NC/GFP expression in the different cell types is shown. MDMs were defined as CD11b^+^, cell lines as CD11b^-^, and phagocytic MDMs as CD11b^+^CTV^+^. Gates were set to determine the frequencies of NC^+^ cells and CTV^+^CD11b^+^ MDMs. The same strategy was applied to cells infected with VSV*ΔG-S21 but with a gate on GFP^+^ cells. A representative graph for data obtained after 24 h of coculture is shown.

**S2. IFN-α and proinflammatory cytokine production in cocultures of pDCs with MDM-φ-A549AT*^inf^*.** MDMs were cocultured with SARS-CoV-2-infected A549AT cells for 2 h. After three washes, pDCs were added, and cocultures were run for an additional 18 h. **(A, B, C)** IFN-α, IL-6 and TNF levels in supernatants determined by ELISA. Labels from the x-axis indicate the cell types present in the cultures. The panels represent data from cells from 3-9 different donors run in single replicates from independent experiments. The results are presented as scatter plots with bars representing the mean ± standard deviation. Statistically significant differences between pDC responses to SARS-CoV-2 and pDCs cocultured with MDM-φ-A549A are indicated by asterisks; differences between pDCs cocultured with MDM-φ-A549A and other groups where cytokines were detected are indicated with asterisks on bars (*p < 0.05, **p ≤ 0.002, ***p ≤ 0.001 and ****p ≤ 0.0001).
